# Mechanisms of the Osteogenic Switch of Smooth Muscle Cells in Vascular Calcification: WNT Signaling, BMPs, Mechanotransduction, and EndMT

**DOI:** 10.3390/bioengineering7030088

**Published:** 2020-08-06

**Authors:** John Tyson, Kaylee Bundy, Cameron Roach, Hannah Douglas, Valerie Ventura, Mary Frances Segars, Olivia Schwartz, C. LaShan Simpson

**Affiliations:** Department of Agricultural and Biological Engineering, Mississippi State University, Starkville, MS 39762, USA; johntyson20@gmail.com (J.T.); kb2978@msstate.edu (K.B.); cpr164@msstate.edu (C.R.); hed165@msstate.edu (H.D.); vcv14@msstate.edu (V.V.); ms3266@msstate.edu (M.F.S.); oschwatrz15@gmail.com (O.S.)

**Keywords:** vascular calcification, smooth muscle cells, canonical WNT, RUNX2, BMPs, integrins, cadherins, EndMT

## Abstract

Characterized by the hardening of arteries, vascular calcification is the deposition of hydroxyapatite crystals in the arterial tissue. Calcification is now understood to be a cell-regulated process involving the phenotypic transition of vascular smooth muscle cells into osteoblast-like cells. There are various pathways of initiation and mechanisms behind vascular calcification, but this literature review highlights the wingless-related integration site (WNT) pathway, along with bone morphogenic proteins (BMPs) and mechanical strain. The process mirrors that of bone formation and remodeling, as an increase in mechanical stress causes osteogenesis. Observing the similarities between the two may aid in the development of a deeper understanding of calcification. Both are thought to be regulated by the WNT signaling cascade and bone morphogenetic protein signaling and can also be activated in response to stress. In a pro-calcific environment, integrins and cadherins of vascular smooth muscle cells respond to a mechanical stimulus, activating cellular signaling pathways, ultimately resulting in gene regulation that promotes calcification of the vascular extracellular matrix (ECM). The endothelium is also thought to contribute to vascular calcification via endothelial to mesenchymal transition, creating greater cell plasticity. Each of these factors contributes to calcification, leading to increased cardiovascular mortality in patients, especially those suffering from other conditions, such as diabetes and kidney failure. Developing a better understanding of the mechanisms behind calcification may lead to the development of a potential treatment in the future.

## 1. Introduction

**Figure 1:**
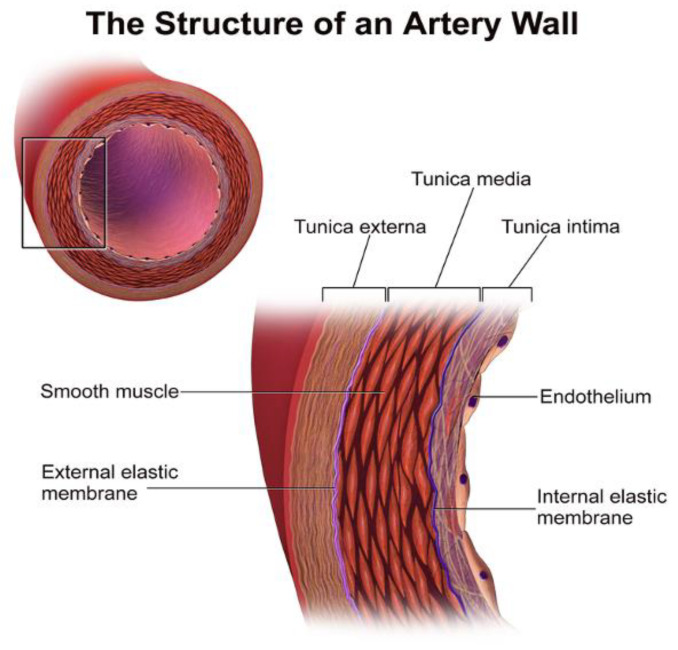
The structure of an artery wall. VSMCs are located in the tunica media, while endothelial cells are mostly in the tunica intima [26].

Mechanical influence over tissue homeostasis is a predominant feature in bone formation and maintenance, acting as a promoter and regulator [1,2]. If the regulatory functions controlling the development of the bone matrix become overwhelmed, such as in the case of tissue injury, mineralization of soft tissue systems becomes a lethal phenomenon, commonly known as ectopic calcification [3]. An ever-increasing prevalence of mineralization is being recognized, specifically in vascular tissues. Vascular calcification is a comorbid pathology alongside obesity, diabetes, and chronic kidney disease. The buildup of hydroxyapatite crystals in various arterial layers, notably the tunica media (Figure 1), promotes hypertension, atherosclerotic plaque burden, and the erosion of arterial tissue compliance and elastance on arteries [4]. There are many regulatory bone formation and structural proteins that are expressed in the calcified medial arterial layers and atherosclerotic plaques, which suggest that this is an active process [5]. The process originates from vascular smooth muscle cells (VSMCs) that have undergone a phenotypic switch into osteoblast-like cells. Unlike other smooth muscle cells, VSMCs can change phenotype due to their plasticity [6,7]. Originating as mesenchymal stem cells, they possess the ability to differentiate into a specific single-lineage based on the induction media [8]. Calcified plaques characterized by differentiated VSMCs within arterial tissues cause a gradual decrease in compliance and subsequently reduce the overall structural integrity of arteries [9,10,11,12]. This reduction is dangerous as arteries are under constant levels of cyclic strain [13,14]. Due to the nature of these consistent levels of strain, it can be inferred that, like bone, arterial tissues respond structurally and chemically to differing levels of stress to maintain homeostasis. For bone, this process involves the mechanically induced deposition of hydroxyapatite crystals throughout the extracellular matrix (ECM), providing a rigid yet durable scaffold [15]. With arterial tissues, strain has shown to promote VSMC proliferation and differentiation [16,17]. In the event of osteoblast-like differentiation, it is suggested that the arterial matrix will be converted into bone-like matrix, forming a region of gradual plaque growth. Under conditions of excessive strain, these regions could begin to grow into calcified plaques, overwhelming regulatory agents. Such agents are interconnected through the canonical WNT signaling cascade, one of the body’s primary structural pathways [18,19,20]. Runt-related transcription factor 2 (RUNX2) is the primary transcription factor responsible for this phenotypic shift and is a target gene of the WNT cascade [21]. This cascade is ubiquitous across the body and evidently controls various structural processes. During WNT-based osteogenesis, studies have demonstrated a link between matrix receptors known as integrins, cell-to-cell receptors known as cadherins, and a set of growth factors known as bone morphogenetic proteins (BMPs) [19,22]. Under stiff matrix conditions and mechanical stress, specifically tension, these proteins potentially synergize with the WNT cascade to induce further osteogenesis through RUNX2 in arterial tissues, possibly increasing calcification [23,24,25]. In addition to VSMCs, the underlying endothelium also contributes to vascular calcification via endothelial to mesenchymal transition. Understanding each of these mechanisms and their role in promoting calcification may help lead to a targeted treatment in the future.

## 2. Phenotypic Switch

**Figure 2:**
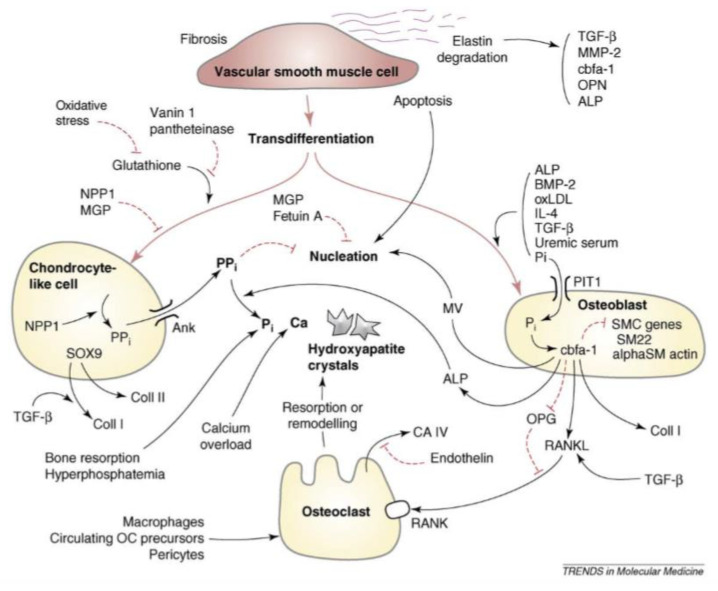
Pathways leading to the differentiation and mineralization of VSMCs to osteoblast-like cells. Factors and mediators shown include transforming growth factor-β (TGF-β), fetuin, hyperphosphatemia, hypercalcemia, bone morphogenetic proteins (BMPs), and nuclear factor-κB ligand (RANKL) [31].

During vascular calcification, VSMCs are believed to undergo a phenotypic switch to osteoblast-like cells. The exact mechanism that initiates the phenotypic switch is unknown, but many studies research the mechanism behind the change to better understand calcification. For example, Essalihi, et al. studied the expression of alpha-smooth muscle actin, tartrate-resistant acid tartrate-resistant acid phosphate, and ED-1 on vascular smooth muscle cells, bone cells, and macrophage phenotypes, respectively [27]. The results showed a decrease in alpha-smooth muscle actin in the calcified VSMCs, supporting the belief that VSMCs undergo a phenotypic switch. In a research study by Patel et al., many similarities and differences between osteoblasts and calcified VSMCs were observed. Osteogenic genes were upregulated in the calcified VSMCs, but the amount of mRNA was much lower than that in the osteoblasts. They concluded that even when calcified, the VSMCs are in the early stages of osteoblast-like differentiation [28]. This transition typically occurs in environments with high serum phosphate and calcium levels. An in vitro study done by C. M. Giachelli showed that inorganic phosphate levels consistent with hyperphosphatemia resulted in increased deposition of calcium in the arteries [29]. In her review, Giachelli explains how increased phosphate levels promote calcification by inducing a phenotypic change in the smooth muscle cells, marked by increased expression of osteochondrogenic proteins [30]. Although the exact mechanism by which calcification takes place is unknown, the common conclusion throughout the literature is VSMCs take on an osteoblast-like phenotype. The transition occurs in the presence of increased calcium and phosphate and is characterized by an increase in osteogenic genes in a process resembling bone formation. This process is tightly regulated by cell-mediated pathways, including the WNT signaling cascade. Figure 2 displays pathways, factors, and mediators for the phenotypic switch of a VSMC.

## 3. Canonical WNT Cascade

**Figure 3:**
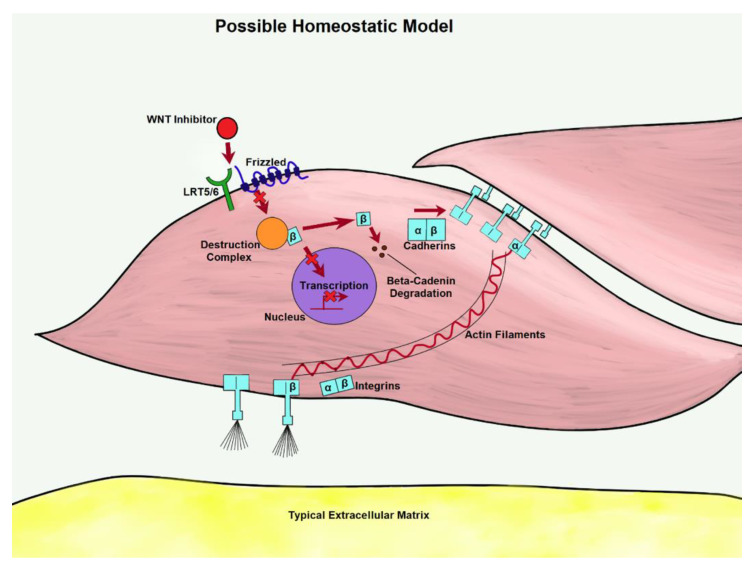
As WNT is inhibited in the homeostatic model, transcription is less likely to take place. β-catenin from disassociated adherins junctions (AJs) could be prevented from pooling through degradation, lowering the expression of matrix proteins.

There are two major WNT pathways that regulate bodily structure: Canonical and noncanonical. Bone formation and osteoblast differentiation is controlled by the canonical WNT signaling cascade, which is dependent on β-catenin. Upon activation of co-receptors frizzled and low-density lipoprotein receptor related protein 5/6 (LRP5/6), a multi-module protein, known as disheveled (DVL), interacts with activated frizzled and recruits the multiprotein “destruction complex” to the cell membrane [32]. This complex is scaffolded by Axin and composed primarily of glycogen synthase 3 (GSK-3), casein kinase 1 (CK1), adenomatous polyposis coli (APC), and β-catenin, a transcriptional coactivator of WNT-targeted genes [33]. Once recruited, Axin binds to LRP5/6, inducing phosphorylation through GSK-3 or CK1, and β-catenin escapes disintegration by translocating to the nucleus. Within the nucleus, β-catenin binds with DNA-binding transcription factors and members of the T-cell factor/lymphoid enhancer factor (TCF/LEF) family to form a complex that promotes specific genes, notably RUNX2 [34]. RUNX2 is a transcription factor required for embryonic bone formation and osteoblast differentiation. In a study done by Gaur T. et al., both in vitro and in vivo experiments showed that WNT signaling induces gene expression of RUNX2 in pluripotent mesenchymal and osteoprogenitor cells [35]. When the expression of RUNX2 is increased in arterial tissues, osteoblast differentiation is promoted among the VSMCs, increasing vascular smooth muscle cell calcification. Inhibitors of the WNT pathway can be used to prevent calcification of VSMCs. Figure 3 displays a possible homeostatic model presenting the effects of the WNT inhibitors and inducers.

A notable inhibitor of the canonical WNT cascade is full-length carboxypeptidase E (F-CPE). F-CPE inhibits the WNT pathway extracellularly. It combines with the frizzled receptor and the WNT3a ligand, forming a complex. It also decreases the expression and activity of β-catenin, which is essential in the WNT cascade [36].

Another protein that can prevent the phenotypic switch is known as sclerostin (SOST). SOST is a protein secreted by osteocytes that acts as a negative regulator of bone formation [36]. It antagonizes the WNT signaling pathway by binding to the LRP5/6 co-receptors and thereby inhibiting the upregulation of RUNX2 [9,37]. It has also been found in postmenopausal women with type two diabetes mellitus, when there are areas of high calcification, there is also an increased level of sclerostin in the calcified areas [38]. A study performed in our lab using an in vitro model of calcified VSMCs, sclerostin expressed the potential to be a therapeutic treatment for vascular calcification by preventing the progression of the WNT pathway [39]. The increased levels of sclerostin also contributed to a decrease in bone mineral density, which is commonly found in patients with vascular calcification [38]. By utilizing sclerostin, the WNT pathway is inhibited, decreasing the osteogenic transdifferentiation of VSMCs.

Inducers of the WNT pathway include the hyperphosphatemia and hypercalcemia, but the splice variant of carboxypeptidase E protein (ΔN-CPE) can also induce WNT. In the study performed by Skalka, ΔN-CPE led to upregulated β-catenin protein and TCF/β-catenin-mediated transcription. The levels increased with each dose of ΔN-CPE [36]. Table 1 displays some inducers and inhibitors of vascular calcification, including those belonging to the WNT pathway. Whether being induced or inhibited, WNT results in either upregulation or downregulation of specific WNT-targeted genes.

## 4. Relevant WNT-Targeted Genes

The WNT pathway promotes calcification by upregulating genes associated with VSMC differentiation (Table 2) [31]. RUNX2 is expressed by WNT in VSMCs under high phosphate conditions and is a key component of osteogenic differentiation of mesenchymal stem cells alongside changes in the VSMC phenotype. Another targeted gene, versican (VCAN) [40], is present in high levels in areas of vascular calcification and most commonly upregulated at the site of vascular injuries [41]. Being a major component of the extracellular matrix (ECM), more prevalent expression of VCAN has been observed in areas of fast tissue growth, suggesting a role in cell proliferation, specifically in arterial tissues. As with most proteoglycans, it plays an anti-adhesive role, promoting cellular migration [42]. Due to the effects of targeted gene osteoprotegerin (OPG), osteoclast differentiation is halted through inhibiting nuclear factor-κB ligand (RANKL) [43], possibly increasing osteoblast favorability in the affected tissues. Also affecting favorability, fibronectin, an integrin ligand, appears in elevated levels in the matrix formed by rapidly calcifying cells, connecting WNT to integrin adherence and matrix conditions [44,45]. As WNT regulates both osteoblast differentiation in arterial matrix and bone formation, developing a better understanding of WNT-controlled bone remodeling may provide insight on its role in calcification.

## 5. Bone Morphogenetic Proteins

**Figure 4:**
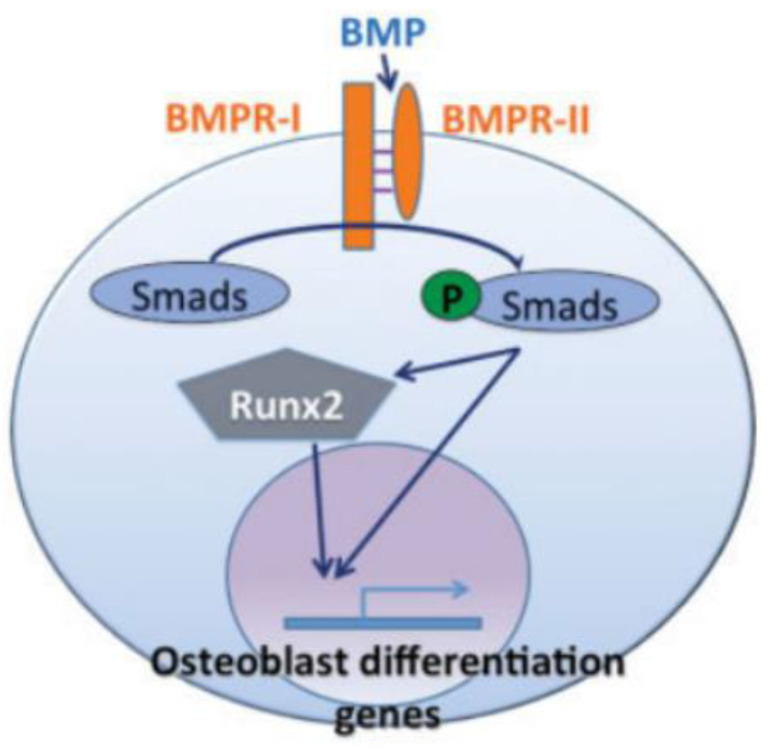
In the BMP pathway, BMP triggers Smad proteins, which then either directly or through RUNX2 transactivate osteoblastogenic genes [22].

Bone morphogenetic proteins (BMPs) are growth factors that play primary roles in bone maintenance and repair. Although examined mainly in bone, BMPs are ubiquitous across the body, playing crucial roles in vascular remodeling. They are multifunctional cytokines and part of the transforming growth factor-β (TGF-β) superfamily. BMP signaling is activated when a BMP binds to a BMP receptor, recruiting an activated quaternary complex. Upon phosphorylation, this complex activates Smad intracellular proteins. When Smad proteins bind to a receptor, they translocate to the nucleus and act as transcription factors, regulating gene expression [22]. This process is displayed in Figure 4.

BMP2 plays the most significant role in vascular calcification, though there are some additional BMPs active in vascular processes. BMP2 expression has been shown by previous studies to be closely associated with the status of osteoblast maturation [18]. Activation of the WNT cascade by WNT3a in osteogenic cells shows an increase in BMP2 expression. BMP2 induces mesenchymal differentiation into osteoblasts, promoting bone formation, and the synergy between WNT and BMP2 holds tight regulation and cooperation between pathways [18]. A study by Hassan et al. indicates that BMP2 activates the transcription of RUNX2 by inducing a homeodomain protein called distal-less homeobox 3 (DLX3), thus forming the mechanism of BMP2 to DLX3 to RUNX2 and vascular calcification [46]. Under the influence of vascular endothelial growth factor (VEGF), WNT3a and BMP4 show synergy, increasing calcification of VSMCs and demonstrating linkage between specific matrix receptors and calcification. Demonstrated through in vitro testing, the knockout of either WNTs or BMPs can potentially inhibit calcification due to the requirement of both for sufficient VSMC mineralization [47]. WNT5a has been shown to increase BMP6 expression, which promotes osteoblastic bone metastases in prostate cancer [48,49]. Responsible for renal tubular differentiation, BMP7 has possible uses as a therapy against atherosclerosis and renal failure. Both WNT1 and WNT11 have activated BMP7 through induction of tubulogenesis [50,51]. BMP9, also thought to affect vascular growth, has been observed to lower VEGF-induced angiogenesis and is highly selective of endothelial cells. This effect ties to the structural function of the WNT signaling cascade. BMP10 expression is associated with overall vascular development in embryos. Both BMP9 and BMP10 have been suggested for treating pulmonary arterial hypertension [52].

## 6. BMPs and Mechanical Stressors

In several studies, high levels of BMP expression have been observed during mechanical stimulation, specifically osteogenesis. Acting as growth factors, BMP2 and 4 seem to work in synergy with immune progenitors in the formation of cartilage and bone. This was demonstrated by Sakoda et al. through the mechanical loading of an osteoblast-like cell line. They found that after prolonged weak loading, osteoblast-like cells expressed high levels of macrophage colony-stimulating factor (M-CSF), a cytokine that is critical for the differentiation of osteoclasts from immune progenitors, and when placed under high loading, they observed high levels of BMP2 and 4 [53]. High levels of BMP2 and 4 were observed during the distraction osteogenesis, a treatment for bone deformation. During the distraction, or load phase, a callous was formed between two slowly separated segments of bone, and BMP2 and 4 were expressed heavily. Following the distraction, the newly formed matrix was calcified, and BMP-6 was lightly expressed [24]. It is suggested by Rui et al. that the cyclic overload of tendon-derived stem cells promotes the formation of calcium nodules through an uptick in BMP2 [54]. Being a structural component that sees both heavy mechanical strain and periods of extended rest, perhaps the results found from the overloaded tendon cells can be extended to cardiovascular tissue as well. Speculated by Balachandran et al., the endothelial layer of porcine aortic valves may act as a mechanosensor under cyclic mechanical strain. Like the bone matrix, porcine aortic valves contain high levels of BMP2 and 4 when in a calcifying state. Valve cusps, thought to be stretched considerably through hypertension, expressed high levels of BMP2 and 4 in vitro and when noggin, an inhibitor for BMP2 and 4, was introduced, the resulting calcification was diminished in a concentration-dependent matter. In a stretch-dependent matter, porcine aortic valve cusp cells under a 10% level of stretch resulted in a low level of apoptosis and at a 15% level of stretch resulted in high levels of apoptosis. When treated with noggin, both levels were also considerably diminished [55]. Seeing the ubiquity of BMP2 and 4 on the calcification of mechanically active tissues, it serves us well to include them in our model.

## 7. WNT Bone Remodeling and Mechanical Strain

Being composed primarily of hydroxyapatite, the bone matrix is a stiffened scaffold that provides structural support and protection. Four cells reside in bone tissue: Osteoblasts, osteoclasts, osteocytes, and bone-lining cells. These cells are involved in an intricate process of remodeling where hydroxyapatite is absorbed by osteoclasts, tissues undergo a transition period, and then hydroxyapatite is deposited by osteoblasts on a type I collagen matrix [56]. Collagen type I plays an important role in the bone matrix, as it acts as a scaffold for the unique calcification of bone. Implicated in the mechanical properties of bone, it provides strength and elasticity throughout bone’s rigid structure [57]. Bone remodeling is primarily activated by either overload or disuse. It follows a U-shaped curve in which the range of typical use is illustrated as the normal physiological window. Within this window, there is low bone turnover [2].

**Figure 5:**
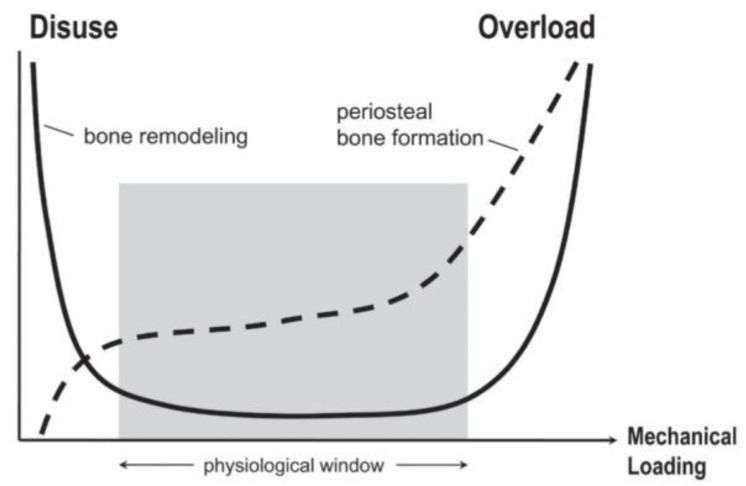
Bone remodeling follows a U-shaped curve and is activated by either disuse or overuse. Typical use between these two extremes is shown by the physiological window, during which there is low bone turnover. In contrast, periosteal bone formation increases directly with an increase in mechanical loading and is low during disuse [2].

During overload, periosteal bone formation occurs at its highest levels, and in both states, bone turnover occurs [2]. Figure 5 displays the U curve of bone formation and remodeling. Under in vitro conditions, osteoblasts exposed to tensile and compressive strain displayed an increase in functionality, as well as a prolonged lifespan. This mechanical stimulation has been shown to function biochemically through canonical WNT responses, notably through WNT10b and coreceptor LRP5 [58]. It has been shown in vivo that bone under dynamic compression creates a promotive effect on the anabolic function of bone remodeling. Although loading did increase growth, the primary occurrence of bone growth occurred after the release of the cyclic load [59,60]. The process of bone remodeling occurs through synergy between the canonical WNT and RANKL pathways [61,62]. WNT activation in osteoblasts has been shown to express OPG, specifically ligand WNT3a. OPG acts as a decoy receptor for RANKL, regulating osteoclast differentiation [63]. As loading reaches a minimal point, osteocytes begin to release the WNT inhibitor SOST, and the removal of damaged bone tissue overtakes the synthesis of hydroxyapatite [64]. As loading is again initiated, WNT is mechanically activated, perpetuating the cycle of loss and growth in terms of bone mass [65]. Acting through the same mechanism bone utilizes for homeostasis, it can be inferred that an increase of compressive and tensile strain will induce bone-like remodeling in the arterial wall. Shown to be present in calcified plaques, osteoclast-like cells are thought to be the missing link between the RANKL/OPG pathway and canonical WNT [66,67]. Shown to interact through osteoblast-induced macrophage differentiation to osteoclasts in vitro, the balance between these two reciprocal pathways is predicted to be disrupted by mechanical overstimulation [68]. As RANKL is deactivated from WNT-expressed OPG, deposited hydroxyapatite overwhelms the immune system’s ability to remove it. Osteoblast-like cells are given a platform for proliferation, and calcified plaques grow larger, further narrowing the artery. This process can be activated or accelerated with excess mechanical stress.

## 8. Mechanical Influence on Arterial Tissues under Pathological Conditions

VSMCs within the tunica media are undifferentiated but can phenotypically shift to osteoblast-like cells once placed under environmental stimulus, such as mechanical stress. Plaque burden changes the morphology of tissues, inducing an environment where strain levels are elevated [69,70,71]. As arteries narrow, the velocity of the blood increases to maintain continual flow. Subsequently, an increase of the velocity creates higher levels of pressure [72,73]. Blood pressure continually places VSMCs under triaxial loading composed of two tensile strains (hoop and axial) and one compressive strain (radial). Under typical conditions, blood pressure stimulates homeostatic responses. However, under hypertension, pressure could potentially induce an excessive mechanical response, exacerbating vascular injury [74,75,76,77]. A combination of both pathologies may overstimulate remodeling responses, and in the case of calcification, promote the growth of the present mineralized matrix. Mechanical activation of WNT has shown to produce a feedback loop in the structural maintenance of vascular tissues, so this mechanical overstimulation may drive the growth of calcified plaques [78,79,80]. VSMCs actively differentiate dynamically to remodel arterial tissues after injury. The variability in VSMC response to tension is due to multiple factors, such as frequency, time, and axial deformation. In a study performed by Laura-Eve Mantella, Adrian Quan, and Subodh Verma at St. Michaels hospital showed how frequency affects VSMC alignment response. A Flexcell system applied vacuum pressure to deform elastomer-bottomed cell culture plates and allowed the user to adjust the frequency, duration, and degree of stretch [14]. They found each factor can cause a response in stretched cells. In a similar study by Liu et al., results indicated stretch-induced alignment was dependent on the frequency of stretch, evidenced by higher alignment perpendicular to the axis of stretch, most effectively at 1.25 Hz. [81]. These results conclude that mechanical stretching induces proliferation and response of VSMCs [14]. In many cardiovascular diseases, it has been found that cyclic stretch evokes not only VSMCs’ proliferation but also migration, phenotyping, apoptosis, phenotypic switching, and vascular remodeling. A study by Liu et al. found that in the presence of angiotensin II (AngII), a hormone that stimulates VSMCs, VSMCs undergoing mechanical stretch significantly upregulated protein expression of AngII type 1 (AT1) receptor, epidermal growth factor (EGF) receptor, and mitogen-activated protein kinase phosphatase-1. In this study, Wistar-Kyoto (WKY) and spontaneously hypertensive rats (SHR) underwent stretch tension by a flex culture system to study the effects on VSMCs from the stretch. They found that mechanical stretch directly regulates AngII-induced smooth muscle cell proliferation and that the AT1/EGF receptor/ERK-dependent signaling pathway is involved in proliferation in smooth muscle of SHR [82]. AT1 can mediate the entry of magnesium by the means of the transient receptor potential melastatin 7 (TPRM7) channel. Low magnesium levels have been associated with vascular calcification and thus a study hypothesized that with the mediation of magnesium Ang II could prevent phosphate-induced calcification [83]. Each of these studies indicate the stretching and mechanical stress can cause a proliferation and varied response of VSMCs. In the vasculature, mechanical stress can result from high blood pressure and other physiological forces.

## 9. Physiological Mechanical Forces

**Figure 6:**
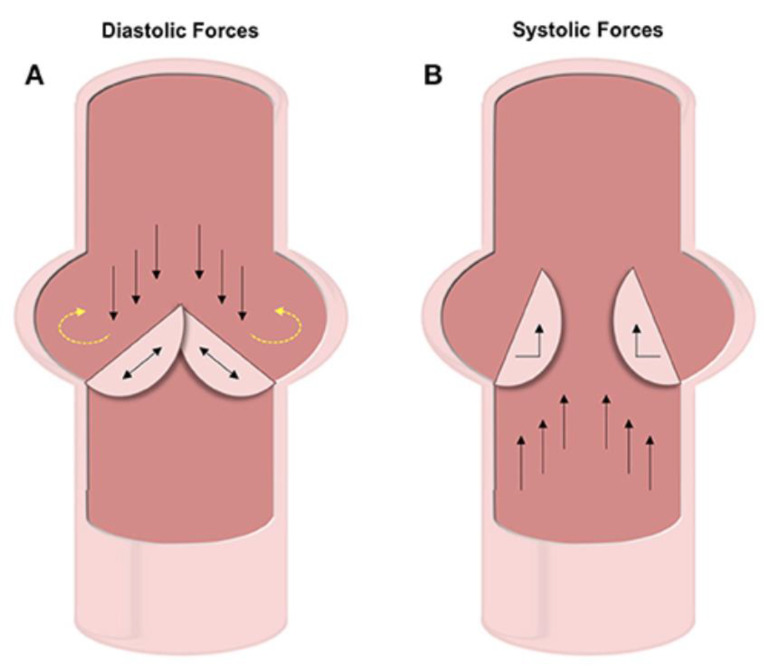
In diastole, as the valves are closed to prevent back flow (black arrows), pressure builds on the valve and circulates onto the wall (yellow arrows). In systole, the valves are forced open by flow propelled by contraction (black arrows) [88].

The cytoskeleton plays a critical role in mechanotransduction. In cardiomyocytes, mechanosensitive proteins in the cytoskeletal network adapt to mechanical stimuli by changing their polymerization states and can thus be translated into functional changes [84]. The aortic valve experiences about 3 billion cycles of opening and closing during the average human lifespan, due to the cardiac cycle involving both systole and diastole. Over the full cardiac cycle, differing mechanical stresses are placed on the ventricles and arteries of the heart. These forces include compression and oscillatory shear on the fibrosa and vascular endothelial cells (VECs) and tensile strain on the vascular interstitial cells (VICs). The fibrosa, spongiosa, and ventricularis are the three main layers of the aortic valve’s dense ECM. VICs are found within each layer, while VECs blanket the entire structure [85]. VECs are mechano-sensitive cells that help the regulation of hemostasis based on mechanical forces. These cells have shown that they can transition from endothelial cells to mesenchymal cells and can thus result in the initiation of calcification [86,87]. The forces on the fibrosa cause calcification and can best be seen in Figure 6 [88].

Vascular regions that branch and curve can experience non-uniform, turbulent flow due to the variety of angles blood must flow through. The flow of the aortic valve is non-uniform because its mechanical environment is cyclic and multidimensional, whereas vasculature experiences more steady uniform flow. These vascular regions are more prone to vascular calcification when compared to those under laminar flow. However, branches that experience laminar flow are consistently undergoing laminar shear stresses. The initiation and progression of vascular calcification in these vascular regions share many similarities to the way bones react under mechanical stimuli. More studies elaborate on the similarities in order to treat and reverse vascular calcification. In a recent in-vitro study by Weinberg EJ et al., a pairing finite-element analysis found that endothelial cells exposed to ventricular-like stresses had higher levels of “atheroprotective” factors than endothelial cells undergoing fibrosa-like stress [89]. Atheroprotective, in this case, means protecting against the formation of atherosclerosis. VSMCs are more prone to injury and calcification due to their physiological design and the forces they encounter.

## 10. Arterial Matrix Stiffness under Pathological Conditions

VSMCs undergo adaptive remodeling based on the levels of cyclic strain they experience. Specifically, the matrix conditions in the medial layer of arteries have been shown to determine the VSMC phenotype in vitro, based primarily on the physical substrate present [90]. A base level of stiffness creates conditions for tissue homeostasis and promotes typical VSMC function. Throughout the cardiovascular system, the arterial cells are constantly experiencing mechanical stresses, as described previously. These stresses can also include transmural pressure, pulsatile pressure, and shear stress. In a review done by Osol G. for the *Journal of Vascular Research*, it was recorded that VSMCs are exposed to multiple mechanical stimuli, such as transmural pressure due to pulsatile pressure and circumferential wall tension [91]. Transmural pressure can be described as the difference of pressure in the lung cavity. However, excessive stiffening caused by disease or aging creates an environment that induces an immune response, such as in the case of atherosclerosis [92,93,94]. Atherosclerosis is distinguished by calcification of the intima layer of the vasculature, resulting in lesions and plaque formation. The intima stiffens alongside the medial layer, but the two process are typically studied independently. Little is known about how the stiffening of other layers affects the vascular adventitia, but a study done by Li et al. provides evidence that calcification can also occur in the adventitia [95]. Cells in the adaptive and innate immune systems are involved in atherogenesis consisting of the stimulation of endothelial cells by adhesion molecules, monocytes and macrophages, dendritic cells, and T and B cells [93,94]. Additionally, some studies have shown that a stiff matrix increases the expression of RUNX2 through the mitogen activated protein kinase/extracellular-signal-regulated kinase (MAPK/ERK) pathway, further leading to cellular calcification [92]. These studies show that matrix characteristics, influenced by excessive mechanical stimuli, can promote a response in the VSMCs leading to calcification.

## 11. Collagen Influence

The ECM is known to have a vital role in the differentiation of cells. In addition to stiffness, the porosity, density, and many other factors can change the way a cell develops within the matrix. Most importantly, these conditions have been identified as promoters of calcification through the influence of collagen [96]. Collagens are ECM proteins that act as possible staging areas for atherogenesis. Cells secrete specific matrix conditions that promote typical cellular function, and collagen I is the most expressed in the body. The most prevalent collagen variants present in the medial layer of arterial tissues is collagen type III alongside collagen type I [96]. Collagen I has been implicated in the promotion of VSMC phenotypic differentiation into osteoblast-like cells. Within bone tissues, osteoblasts produce hydroxyapatite crystals along collagen I, mineralizing it; this process is present within arterial tissues through osteoblast-like cells exhibiting osteogenic functions [97,98]. Under atherosclerotic conditions, the total content of collagen type I increases [99]. Like bone mineralization, the increased collagen I content in the matrix promotes deposition of hydroxyapatite along the arterial wall. Collagen type II, typically associated with ossification in specific bone structures, has been found to congregate in high concentrations in response to elevated levels of cholesterol and increased intensity of atherosclerotic calcified plaques [100]. The matrix produced by rapidly calcifying VSMCs accelerates the mineralization rate of transplanted cells. This matrix is composed of three times the typical collagen I and fibronectin content. The total collagen type IV levels are lowered by 70% to that of non-mineralizing cells, implying that collagen type IV inhibits mineralization. To support these findings, slowly mineralizing clones placed on a pure matrix of either collage type I or fibronectin showed an increased rate in mineralization. However, the mineralization process of clones placed on a matrix of collagen type IV was significantly inhibited [45]. With the considerable differences found between collagen types and the resulting cellular response, it is suggested that the ECM will propagate the conditions necessary for calcification and be the staging area for mechanical influence through ECM to cell communication.

## 12. Integrins and Relevant Functions

**Figure 7:**
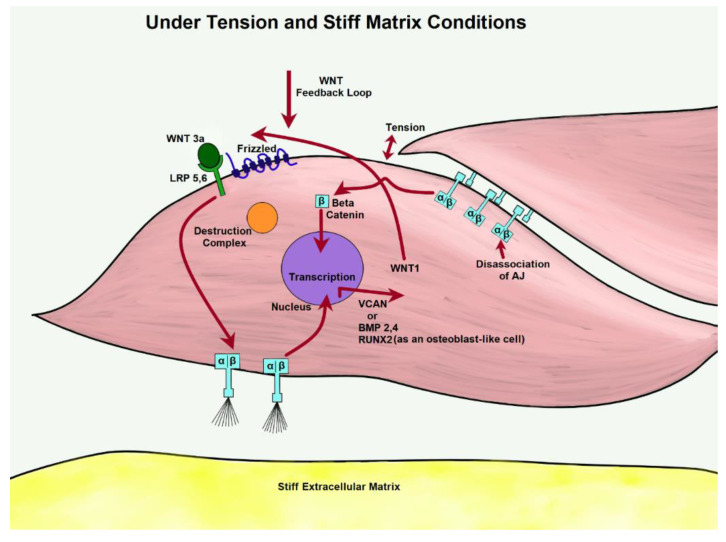
Through the activation of WNT, β-catenin is free to pool from tension-dissociated AJs. Under excessive tension, integrins are likely to influence WNT expression of matrix proteins.

Integrins are transmembrane receptors that can act as the primary matrix anchors that bind to extracellular membrane proteins. They serve to mediate cell adhesion and interpret mechanical signals from the ECM. They are formed through noncovalent association of two sub-units, α- and β-, and attach to components, such as laminin, collagen, and fibronectin. Integrins recognize several different matrix ligands, allowing for a variety of cellular functions in terms of adhesion and cellular motility. Adhesion, responsible for linking extracellular substratum to actomyosin, regulates signaling pathways that promote proliferation, gene expression, and cell survival. Environmental forces, such as mechanical stress and matrix stiffness, influence adhesion-based integrin function due to linkage with actin [101]. In response to collagen I ligation, integrin activation cascades to the focal adhesion kinase (FAK) pathway, leading to β-catenin translocation and enhanced transcription [102]. Reportedly, the WNT variant WNT3a greatly increases adhesion to collagen type I through the upregulation of integrin-linked kinase (ILK) and subsequent activation of the β-1 subunit, the primary adhesion protein of VSMCs [103]. Activated on a stiff matrix, integrins α1β1 and α10β1 phosphorylate GSK3 through the ERK/FAK pathway, preventing β-catenin degradation in osteoblastic cells. Translocating to the nucleus, β-catenin binds to the target gene WNT1, creating a positive feedback loop [25]. Figure 7 below shows a possible homeostatic model of this process.

## 13. Integrins and Mechanical Stressors

Integrins interpret mechanical signals from the ECM in a linear fashion, possibly regulating signal pathways in cells. After initial activation by respective ligands, integrins attach to the actin cytoskeleton, increasing cell adhesion [104]. Cells adapt physically to integrin-applied forces as well as maintain structural stability and protect the body from mechanical injury. It is speculated that variations in mechanical strain, from static to dynamic, produce different biochemical cues [105]. Under mechanical strain, fibronectin and vitronectin elicit high activation of several integrins. Additionally, the inhibition of the β-subunit seems to only partially lower the strain response. In rat VSMCs, sub-unit αV is suggested to strongly interpret mechanical strain [106]. An in vitro study using isolated arterioles and VSMCs found that integrins may have crucial mechanotransductive elements for VSMCs. The study demonstrated that the mitogenic responses of VSMCs under strain were dependent on the composition of the ECM to which it adheres [106]. VSMCs on fibronectin had the most significant mitogenic response to strain because of the increased integrin binding. Another recent study argued that the machinery of adhesion in multicellular tissues is an interdependent network of cell-to-cell and cell-to-ECM interactions and signaling responses, as opposed to crosstalk between spatially and functionally distinct adhesive tissues within the cells [107]. Both have an essential role in the way the cell responds to changes. In the adhesive state, they are altered through physical changes with the cytoskeleton and by participating in multidirectional cell signaling events [23]. These complexities were found to be part of a larger adhesive network where multiple types of cell adhesions occur and interact. Integrins have been closely tied to cadherin function, as both are sensitive to mechanical stress and modulate through matrix stiffness [108]. The network formed between cadherins and integrins is essential to the cell signaling pathways and cytoskeletal assemblies involved in the regulation of cell polarity, migration, proliferation and survival, differentiation, and morphogenesis.

## 14. Cadherins and Relevant Functions

Cadherins function as cell-to-cell adhesion receptors and play crucial roles in tissue morphogenesis and homeostasis. When cells are in close contact, opposing cadherins bond and regulate further contact. As cadherins bond, they form a zipper-like structure and tie cells together. Once cells are bound together, the overall adhesion tension is lowered, and further interfacial tension is regulated. Cadherins are anchored to the cytoskeleton through actin binding proteins and mediated by α- and β-catenin [109]. Adheren junctions (AJs) are formed by bound cadherins through cell-to-cell contact. Their primary function is to maintain physical association between cells. Disruption of this association, such as through tension, causes cell-to-cell detachment and disorganizes tissues [110]. Dissociation of AJs has been linked to an increase in β-catenin pooling and subsequent transportation into the nucleus. This suggests integration between cadherin and WNT activation, producing supplemental gene transcription [111]. Because integrins and cadherins activate many of the same signaling pathways, many consider them to be interdependent functional nodes. However, instead they should be considered as functionally equivalent. A single node can influence adhesion function and signaling activities of adhesion.

## 15. Cadherins and Mechanical Stressors

Cadherins sense mechanical changes through F-actin association. Their junctions adapt to both internal and external forces including matrix stiffness. Fluctuations sensed by cadherins induce biochemical responses that alter junction properties and biological processes, such as gene transcription [112]. Tied through the actin-myosin cytoskeleton, cadherins and integrins form adhesive networks that modulate intracellular tension in response to intercellular tension sensed by FAKs and AJs. FAK induces dissociation of β-catenin from VE-catenin in a tension-independent manner. The FAK pathway also increases N-cadherin expression in mouse VSMCs, coinciding with an increase in VSMC proliferation under arterial stiffening. Mechanical stress placed on a cell is shared simultaneously with both cadherins and integrins, suggesting a homeostatic relationship. As the strengthening of one adhesion receptor type increases, the other type decreases [113]. Integrins and cadherins are further connected through mechanisms of differentiation in bone [114]. Integrin signaling has been found to promote a positive feedback loop in bone morphogenetic proteins [115], as cadherins also play a crucial role in BMP2-induced osteoblast differentiation [116].

## 16. Seeding through Thrombus Release and Other Health Risks

**Figure 8:**
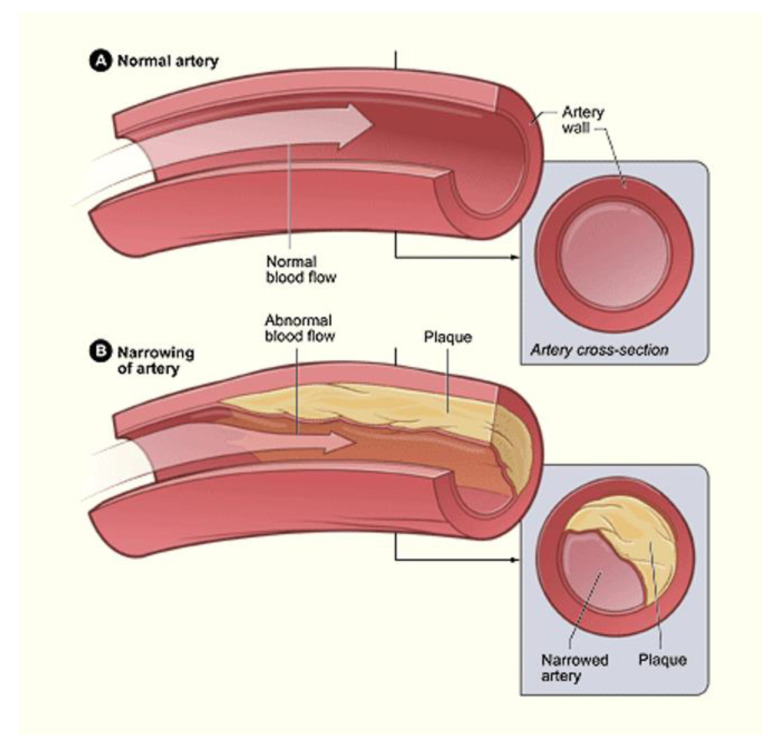
Although able to exist singularly, lipid-based plaques and calcified plaques are often complementary in the narrowing of arteries. This narrowing is illustrated through the contrast of the normal artery (**A**) and the narrow artery (**B**) [122].

As each of the previously described factors contribute to growing calcified plaques, the susceptibility of plaque rupture increases. Once a plaque ruptures, the thrombus produced can travel to healthy tissues, attach, and restart the cycle of lesion creation to plaque development [117,118,119]. If this is the case, then the mechanically stimulated development of calcified plaques is comparable to cancer, and treating it becomes that much more difficult. Because of this, vascular calcification greatly increases the cardiovascular mortality rate of patients, especially those with other underlying conditions. Diabetes patients are more than twice as likely to experience cardiovascular mortality, compared to nondiabetic individuals. Vascular calcification has been used as an early indicator for those patients of high risk [120]. Diabetes is also known to be the main cause of end-stage renal disease. Cardiovascular disease is the leading cause of death for dialysis patients, and, on average, 83% of dialysis patients have some level of coronary artery calcification [121]. Figure 8 is a representation of calcification in an artery.

A study done by Patel et al. showed that calcifying VSMCs have higher rates of apoptosis and lower cellular viability than control VSMCs and osteoblasts [28]. The study showed how calcification damages VSMCs and reduces compliance and structural integrity, leading to heart attack or stroke. Developing a better understanding of the mechanisms behind calcification and its similarities to bone formation may allow for the creation of a target treatment to prevent or decrease vascular calcification, particularly among these high-risk groups. Alongside biochemical treatments, changing the patient lifestyle could lower the rate of calcification. Examples of such changes include weight loss and a balanced diet, which can aid in reducing hypertension and lowering mechanical strain on vascular walls. These lifestyle choices can either increase or decrease the risk of diabetes, kidney disease, and cardiovascular mortalities.

## 17. Endothelial to Mesenchymal Transition (EndMT)

**Figure 9:**
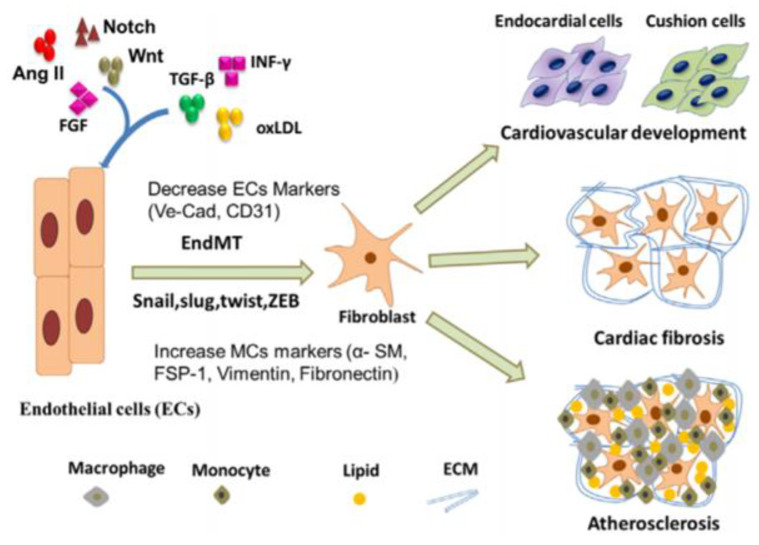
The process of endothelial cells (ECs) differentiating into fibroblasts, leading to cardiovascular development, cardiac fibrosis, or atherosclerosis [124].

In congruence with smooth muscle cells and their role in vascular calcification, endothelial cells are also seen to play a major role in the deposition of minerals in the arteries. This relatedness could be due to their proximity to each other in the arteries, with smooth muscle cells primarily located in the tunica media of the arteries, and endothelial cells found primarily in the tunica intima of the arteries. The specifics of vascular calcification in relation to endothelial cells involves the process of endothelial to mesenchymal transition (EndMT). EndMT is a process in which endothelial cells lose their genetic markers, VE-cadherin, and CD31, and begin to obtain the phenotype of mesenchymal cells through the expression of mesenchymal cell markers, such as α-smooth muscle actin, fibroblast-specific protein 1 (FSP-1), and fibronectin [123]. Endothelial cells, primarily found in the tunica intima, are known to prevent cardiovascular diseases, so when they differentiate into mesenchymal cells via EndMT, it gives rise to various cardiovascular diseases. EndMT has been shown to cause vascular calcification, a common complication of the end stage of atherosclerosis. Vascular calcification is a regulated process of mineralization in the arteries involving osteoblasts forming from EndMT. This process resembles that of bone mineralization and was previously believed to be a factor of aging but is now recognized to be potentially preventable. Vascular calcification is typically seen to occur in the medial or intimal layers of the blood vessels, with the medial layer also being associated with smooth muscle cells and chronic kidney disease, diabetes, and hypertension, and the intimal layer typically being associated with endothelial cells and their role in atherosclerosis and blood clots. However, the exact mechanisms and processes through which EndMT occurs are still unknown. The processes discussed are seen in Figure 9.

## 18. EndMT in TGF-β and BMP Signaling Pathways

Recently, various studies have shown that the overexpression of TGF-β/smad signaling and bone morphogenic protein (BMP) signaling, both belonging to the TGF-β superfamily, contribute to EndMT vascular calcification [123,124,125,126]. TGF-β is a cytokine that plays various roles in embryogenesis, cellular development, and homeostasis. In the TGF-β/smad signaling pathway, the overexpression of TGF-β promotes endothelial cell dysfunction and the generation of myofibroblasts. The endothelial cell dysfunction can occur due to TGF-β activating EndMT via smad2, smad3, and smad4, forming a complex that suppresses the VE-cadherin marker, leading to the occurrence of EndMT [124]. Conversely, smad7 exerts opposite effects on TGF-β to inhibit the occurrence of TGF-β-induced EndMT [126]. Overexpression of BMP2 induces EndMT, causing increased vascular calcification [126]. BMP is regulated through the matrix Gla protein (MGP), which is primarily induced in atherosclerotic lesions to aid in the reduction in the amount of BMP expressed. MGP binds to BMP2, BMP4, and BMP7 to inhibit their activity, but not enough MGP is induced to overcome the amount of BMP, and thus inhibit EndMT. In instances where MGP is not properly expressed, it can lead to excess BMP activity and subsequently, apoptosis or excessive calcification in the cells [125]. Previous studies noted that vascular calcification might also arise from the potential imbalance between endothelial and smooth muscle cells, facilitated by the TGF-β and BMP signaling pathways.

## 19. EndMT in WNT Signaling Pathway

**Figure 10:**
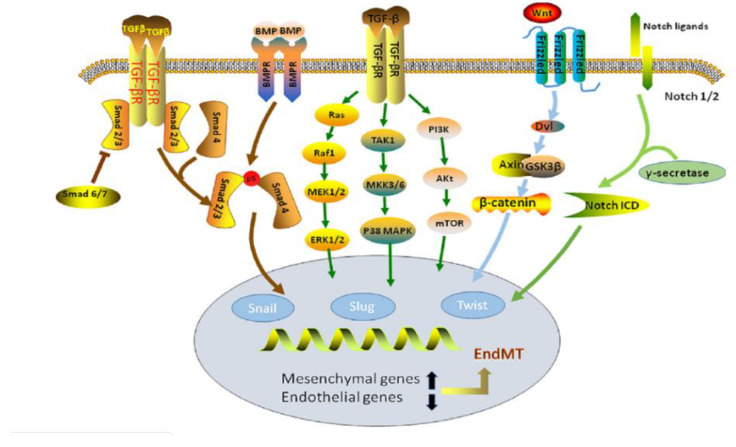
Representation of the TGF-β, BMP, and WNT signaling pathways. The processes that lead to endMT through each pathway are displayed [126].

Another prevalent pathway of EndMT involves the expression of WNT protein as a source of activation of the WNT signaling pathway. The various ligands involved in this pathway influence and conduct cell transformation, cell proliferation, and apoptosis during tissue homeostasis. These ligands include frizzled (Fz), LRP, glycogen synthesis kinase-3β, and β-catenin [127]. The specific mechanism of activation involves the accumulation of β-catenin due to the binding of Fz and LRP receptors to WNT protein. Once activated, WNT signaling induces an EndMT transcription factor known as TWIST (Figure 10). When TWIST is suppressed, EndMT is inhibited. However, the overexpression of TWIST can lead to the promotion of EndMT. The excessive secretion of WNT can stimulate fibroblast production, leading to cardiac fibrosis and calcification. Without a WNT ligand, this pathway enters a “silent” stand, thus inhibiting EndMT [124].

## 20. Calcification of Mechanically Active Vascular Devices

Devices in mechanically active vascular regions are notorious for stiffening and failing over time due to mineralization. It is suggested that the immune system acts as the initiator of calcification through rejection of the device’s material, residual antigens, and the subsequent tearing the device endures. Implanted valves, regardless if based on natural or artificial scaffolding, undergo considerable degradation that impacts their ability to ensure proper blood flow. In younger individuals, calcification of heart valves is higher than their elder counterparts. Considering the higher levels of immune activity in younger people, the increased reaction to the device and following mineralization is expected [128]. Vascular stents fail due to tearing and lesion formation promoting degradation through mineralization. In a numerical study, vascular stents are modeled as a 3-D non-homogenous mesh. During computation of an angioplasty with stenting, simulated tears in the intima altered the stress carried by the layers of the vessel. Normally, all three layers would maintain the load, but as the tears formed from surgical trauma, the tears would endure significant amounts of strain. For a stent to maintain its position and effectiveness, it must place stress proportional to the size of the lumen relative to the size of the plaque. This has led to a tradeoff between the lumen diameter and potential lesion creation during insertion [129]. Due to the need of surgical revision later in a device’s life cycle, efforts have been made to either develop devices out of biocompatible materials or coat the devices in a mineralization resistant chemical [128]. If we develop an understanding behind the promotion of calcification of an implant and the mechanotransduction it undergoes, then perhaps the need for surgical revision can be limited. Ultimately, the body’s reaction to implanted vascular devices demonstrates potential synergy between the initial immune response and mechanical perpetuation of mineralization.

## 21. Conclusions

As calcification mirrors bone growth, the understanding of how mechanical stimuli influences the growth of plaques becomes critical. Knowing how VSMCs respond to their environment could shed light on how osteogenic promoters are expressed. Vascular systems are structurally homeostatic to preserve proper blood flow to tissues. To maintain arterial structure, VSMCs seem to adapt to even minor mechanical stimuli and promote complementary matrix conditions [130]. The excessive mechanical stress seems to promote osteogenic differentiation, though the exact mechanism is inconclusive. However, as structural cascades and adherence proteins are linked together, a bigger picture is painted. This review focused primarily on the WNT signaling cascade, though other pathways are also being explored. As pathological conditions give rise to higher rates of vascular strain, knowing how the reciprocal pathways, particularly WNT and RANKL/OPG, interact in detail could illuminate new methods for treatment. Under typical homeostatic conditions, proliferation and differentiation of VSMCs is lowered alongside gene transcription, whereas in conditions of stiffened extracellular matrix and tension, matrix proteins, such as VCAN, are expressed, promoting matrix contractility [131].

Many studies are trying to understand why VSMCs begin to differentiate and express RUNX2 and BMP2,4 instead. The knowledge underlying the initial conditions is not yet explained, but stiffening matrix conditions alongside excessive cyclic tension could elaborate on the growth of plaques. Once VSMCs are differentiated, the osteoblast-like cells begin altering the surrounding ECM, laying the foundation for mineral deposition [132]. This matrix stiffening may promote further proliferation of VSMCs and subsequent differentiation, repeating the process through the WNT cascade, integrins, and cadherins. Under initial activation through WNT3a, integrin’s influence through β1 weakens cadherin strength. This causes dissociation of AJ, and β-catenin begins to pool. Integrins are then able to influence transcription of WNT1, creating a feedback loop. This feedback loop potentially propagates osteogenic proteins after VSMCs have undergone phenotypic differentiation.

Once VSMCs are differentiated into osteoblast-like cells, BMP2,4 and RUNX2 are expressed. This potentially leads to an increased presence of plaque as the matrix conditions are altered. If the cycle is validated, new therapeutic targets are realized. Instead of simply knocking out the WNT cascade, altering or inhibiting the conditions that influence expression could prevent further mineral buildup. This would allow for minimal side effects while promoting the body’s natural ability to remove foreign material from arterial tissues. Pairing this with changes in patient lifestyle could significantly lower the rate of vascular calcification.

## Figures and Tables

**Table 1:** Vascular calcification regulatory agents, including known inducers and inhibitors.

Inducers	Inhibitors
N-carboxypeptidase E	F-Carboxypeptidase E
Hyperphosphatemia	Sclerostin
Hypercalcemia	MGP
BMP-2 and BMP-4	OPG
RUNX2	Collagen type IV
Injury and stress	

**Table 2:** WNT pathway target genes and the effect of each on the cell.

Gene	Effect
RUNX2	osteogenic differentiation
RANKL	Recruitment of osteoblast-like cell precursors
OPG	regulates bone turnover, blocks RANKL
VCAN	cell proliferation and migration

## Glossary

**Abbreviation**	**Definition**
AJ	adherens junction
BMP	bone morphogenic protein
DLX3	distal-less homeobox 3
EC	endothelial cell
ECM	extracellular matrix
EndMT	endothelial to mesenchymal transition
ERK	extracellular signal-regulated kinase
FAK	focal adhesion kinase
F-CPE	Full length Carboxypeptidase E
GSK-3	glycogen synthase kinase-3
LEF	lymphoid enhancer factor
LRP	lipoprotein receptor-related protein
MAPK	mitogen activated protein kinase
M-CSF	macrophage colony-stimulating factor
MGP	matrix gla protein
OPG	osteoprotegerin
RANKL	nuclear factor-κB ligand
RUNX2	runt related transcription factor
SOST	sclerostin
TCF	T-cell factor
TGF-β	transforming growth factor-β
VCAN	versican
VEC	vascular endothelial cells
VEGF	vascular endothelial growth factor
VIC	vascular interstitial cells
VSMC	vascular smooth muscle cell
WNT	wingless-related integration site
ΔN-CPE	splice variant of carboxypeptidase E
